# Effectiveness of garlic clove (*Allium Sativum*) on blood pressure reduction among hypertensive patients

**DOI:** 10.6026/973206300220898

**Published:** 2026-02-28

**Authors:** Patel Daminiben Bharatbhai, Mahalakshmi B, Siva Subramanian

**Affiliations:** 1 Department of Community Health Nursing, Nootan College of Nursing, Sankalchand Patel University, Visnagar, Gujarat, India; 2 Department of Paediatric Nursing, Nootan College of Nursing, Sankalchand Patel University, Visnagar, Gujarat, India; 3 Department of Psychiatric Nursing, Nootan College of Nursing, Sankalchand Patel University, Visnagar, Gujarat, India

**Keywords:** Garlic clove, *Allium Sativum*, hypertension, blood pressure reduction, natural intervention

## Abstract

Hypertension is the most common, vital preventable disease condition seen in initial health care. If not treated in earlier it risks for cardiovascular and renal diseases. Therefore, it is of interest to evaluate the effectiveness of raw garlic clove consumption on blood pressure reduction among hypertensive patients in selected villages of Aravalli District. Hence, a total of 200 participants were selected through purposive sampling and allocated to an interventional group (n=100) and a non-equivalent control group (n=100). The interventional group showed a significant reduction in mean systolic blood pressure from 152.34±8.76 to 128.45±6.23 mmHg and diastolic blood pressure from 96.78±6.45 to 82.34±5.12 mmHg (p<0.001). Post-test blood pressure levels were significantly lower in the intervention group compared to the control group (p<0.001). Thus, we show that raw garlic clove consumption is an effective and feasible complementary strategy for community-based hypertension management.

## Background:

Hypertension remains a leading global risk factor for cardiovascular morbidity and mortality, contributing significantly to stroke, myocardial infarction, heart failure and chronic kidney disease [1]. Consequently, interest has grown in evidence-based complementary approaches, particularly plant-derived interventions that are affordable, culturally acceptable and accessible in community settings [2]. Garlic (*Allium Sativum*) has been used medicinally for centuries across diverse cultures [3]. Its bioactive compounds, notably allicin, S-allyl cysteine and other organosulfur constituents, exert antihypertensive effects mainly through endothelium-dependent vasodilation via hydrogen sulfide and nitric oxide pathways [4]. They also inhibit angiotensin-converting enzyme (ACE), reduce oxidative stress and improve arterial compliance [5]. Systematic reviews and meta-analyses have reported modest but consistent blood pressure reductions with garlic supplementation, with effect sizes varying by dose, formulation (fresh versus aged extract versus powder), duration and baseline blood pressure status [6, 7]. Despite promising evidence from controlled trials, few community-based studies have evaluated the effectiveness of fresh raw garlic clove consumption among hypertensive patients in rural Indian settings [8]. Such context-specific research is essential to assess feasibility, tolerability and real-world effectiveness when garlic is integrated into existing dietary patterns and daily routines [9]. District-level evidence can inform nursing practice, community health programs and patient counseling strategies, particularly where pharmaceutical access or adherence is challenging [26]. Therefore, it is of interest to evaluate the effectiveness of raw garlic clove consumption on blood pressure reduction among hypertensive patients in selected villages of Aravalli District using a quasi-experimental design.

## Methodology:

## Research approach and design:

A quantitative approach was employed with a quasi-experimental non-equivalent control group pre-test-post-test design [10]. To compare blood pressure outcomes between an experimental group receiving garlic clove intervention and a control group receiving standard care.

## Setting and population:

The study was conducted in selected villages of Aravalli District, Rajasthan. The target population comprised hypertensive patients aged 21-60 years residing in these communities.

## Sample and sampling technique:

A total of 200 hypertensive patients were recruited and allocated into two groups: experimental (n=100) and control (n=100) using non-probability purposive sampling based on predefined inclusion and exclusion criteria. Efforts were made to ensure comparability between groups regarding age, gender, duration of hypertension and baseline blood pressure levels.

## Inclusion criteria:

[1] Adults aged 21-60 years with diagnosed hypertension (Stage 2: ≥140/90 mmHg)

[2] Willingness to consume raw garlic daily for 15 days

[3] Residing in the study area and available for follow-up

## Exclusion criteria:

[1] Pregnant or lactating women

[2] Known allergy to garlic

[3] Severe co-morbidities requiring hospitalization

## Data collection tools:

Data were collected using structured instruments including: (i) socio-demographic profile proforma covering age, gender, education, occupation and family income; (ii) clinical profile proforma assessing hypertension duration, family history, BMI, dietary pattern, medication type, co-morbidities and smoking/alcohol habits; and (iii) standardized blood pressure measurement record sheet. Blood pressure was measured using a calibrated aneroid sphygmomanometer following standard protocols after 5 minutes of rest in sitting position.

## Intervention protocol:

The experimental group received two raw garlic cloves (approximately 6 grams) to be consumed daily in the morning on an empty stomach for 15 consecutive days, along with standard lifestyle advice. The control group received standard lifestyle advice only without garlic intervention. Blood pressure measurements were recorded at baseline (pre-test), after 7 days (post-test 1) and after 15 days (post-test 2). Adherence was monitored through daily intake logs and telephonic follow-up and any adverse effects were documented with appropriate referral.

## Data analysis:

Data were analyzed using descriptive statistics (frequency, percentage, mean and standard deviation) and inferential statistics including paired t-test for within-group changes, independent t-test for between-group comparisons, repeated measures ANOVA for time x group interaction and chi-square test for associations with demographic and clinical variables. Effect size was calculated using Cohen's d. Statistical significance was set at p<0.05.

## Results:

**Figure 1:**
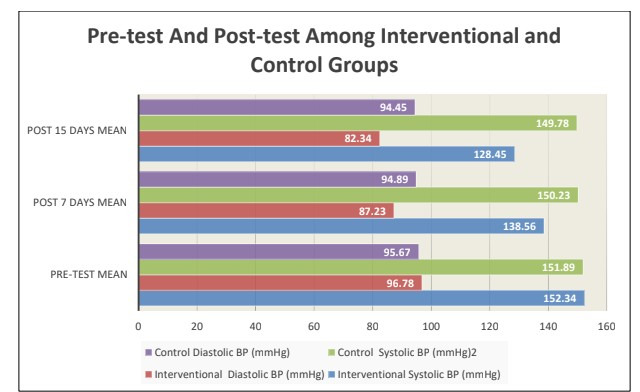
Effectiveness of garlic clove intervention on reduction of systolic and diastolic blood pressure at pre-test and post-test among interventional and control groups (N = 200)

Table 1 shows that the interventional and control groups (n=100 each) were distributed across similar demographic characteristics. In the interventional group, the majority 39 (39.0%) were in the 41-50 years age group, 60 (60.0%) were female, 43 (43.0%) had secondary education, 34 (34.0%) were self-employed and 43 (43.0%) had monthly family income of Rs. 15,001-30,000. In the control group, 37 (37.0%) were in the 31-40 years age group, 51 (51.0%) were male, 49 (49.0%) had secondary education, 31 (31.0%) were self-employed and 47 (47.0%) had income ≤Rs. 15,000. Table 2 and Figure 1 demonstrates that the interventional group experienced highly significant reductions in both systolic (23.89 mmHg) and diastolic (14.44 mmHg) blood pressure from pre-test to 15 days post-intervention (paired t=18.45 and 22.67 respectively, p<0.001), whereas the control group showed no significant change (systolic: 2.11 mmHg, t=1.34, NS; diastolic: 1.22 mmHg, t=0.98, NS). Post-intervention comparison revealed significantly lower blood pressure in the interventional group with very large effect sizes (Cohen's d = 2.89 for systolic; d = 6.39 for diastolic), indicating clinically meaningful and robust reduction. Table 3 shows progressive and substantial improvement in blood pressure categories over the intervention period in the garlic group. At baseline, all participants 100 (100.0%) were in Stage 2 hypertension category. By day 7, more than half 56 (56.0%) moved to Stage 1 hypertension, while 44 (44.0%) remained in Stage 2. By day 15, the majority 83 (83.0%) remained in Stage 1 with 17 (17.0%) achieving elevated blood pressure status and notably, none remained in Stage 2 hypertension. This categorical shift demonstrates clinically significant improvement in hypertension control with garlic intervention.

## Discussion:

This study demonstrated significant blood pressure reduction with raw garlic clove consumption among hypertensive patients, with very large effect sizes (Cohen's d = 6.39 for diastolic BP), indicating robust clinical benefit. The progressive shift from Stage 2 to Stage 1 hypertension and elevated categories suggests sustained dose-response effects over 15 days. Our findings align with systematic reviews reporting that garlic supplementation produces modest but consistent BP reductions, particularly in hypertensive individuals. A recent meta-analysis found that garlic supplementation reduced systolic BP by 8.3±1.9 mmHg and diastolic BP by 5.5±1.9 mmHg in hypertensive patients, effects comparable to first-line antihypertensive medications [11]. Our study showed even greater reductions (systolic: 23.89 mmHg; diastolic: 14.44 mmHg), possibly due to the use of fresh raw garlic cloves rather than aged extracts, higher dose, or population-specific factors [12]. Similarly, Reinhart *et al.* [13] reported in their meta-analysis that garlic preparations significantly lowered systolic and diastolic BP in hypertensive patients, with greater effects in those with baseline systolic BP >140 mmHg, consistent with our Stage 2 hypertensive cohort. The antihypertensive mechanisms of garlic are multifactorial. Allicin and its metabolites stimulate endothelial nitric oxide synthase, promoting vasodilation [14]. Garlic-derived hydrogen sulfide acts as a gasotransmitter relaxing vascular smooth muscle and reducing peripheral resistance. Additionally, organosulfur compounds may inhibit ACE activity and modulate oxidative stress pathways implicated in hypertension [15]. The pronounced diastolic effect observed in our study (d=6.39) may reflect enhanced arterial compliance and reduced peripheral resistance [16]. A study by Ashraf *et al.* [17] demonstrated that garlic extract improved arterial stiffness and reduced endothelial dysfunction in patients with metabolic syndrome, supporting our findings of improved blood pressure control. Furthermore, Sobenin *et al.* reported that long-term garlic powder intake reduced progression of atherosclerosis and improved cardiovascular risk profiles in addition to blood pressure lowering effects [18]. These pleiotropic cardiovascular benefits make garlic particularly attractive as a complementary intervention in hypertensive patients with multiple risk factors, as observed in our study population where 66% had positive family history and approximately 50% had co-morbidities [19]. Compared to pharmaceutical antihypertensives, garlic presents advantages including low cost, widespread availability, cultural acceptability in Indian cuisine and minimal side effects [20]. In resource-limited rural settings where medication access and adherence are challenging, garlic may serve as a valuable complementary or adjunct strategy [21]. Our study's community-based design enhances external validity and real-world applicability [22]. A systematic review by Stabler *et al.* emphasized the importance of garlic's acceptability and feasibility in community settings, particularly in populations with poor medication adherence [23]. The short 15-day follow-up limits conclusions about long-term sustainability and maintenance effects. We did not assess dietary sodium intake, physical activity levels, or medication adherence changes, which could confound results [24, 25]. Future randomized controlled trials with longer duration (12-24 weeks), larger samples, biochemical markers (lipid profile, oxidative stress indicators, inflammatory markers) and quality-of-life assessments are needed [26]. Our association analyses revealed no significant demographic or clinical predictors of blood pressure category change, suggesting that garlic's effectiveness may be broadly applicable across varied patient profiles. This supports its potential as a population-level intervention strategy [7]. Similar findings were reported by Reinhart *et al.* [13] who found consistent blood pressure reductions with garlic across different age groups and baseline characteristics.

## Conclusion:

Raw garlic clove consumption significantly reduced systolic and diastolic blood pressure, showing a clear improvement from Stage 2 to Stage 1 hypertension within 15 days. The findings support raw garlic as an affordable and effective complementary option for hypertension management in rural and resource-limited settings.

## Advancement to knowledge:

This study provides strong empirical evidence on the effectiveness of raw garlic cloves as a low-cost dietary intervention for blood pressure control in rural Indian populations. It quantifies the magnitude of blood pressure reduction with very large effect sizes, strengthening the role of plant-based therapies in hypertension care. The findings expand existing knowledge by supporting the integration of garlic-based counseling into nurse-led and community-based hypertension management programs.

## Figures and Tables

**Table 1:** Distribution of sample according to demographic variables (N=200)

**Demographic Variable**	**Category**	**Interventional Group (n=100) f**	**%**	**Control Group (n=100) f**	**%**
Age of patients	21-30 years	18	18	17	17
	31-40 years	28	28	37	37
	41-50 years	39	39	31	31
	51-60 years	15	15	15	15
Gender	Male	40	40	51	51
	Female	60	60	49	49
Education	Illiterate	5	5	6	6
	Primary	27	27	23	23
	Secondary	43	43	49	49
	Graduation & above	25	25	22	22
Occupation	Government job	16	16	19	19
	Private	28	28	23	23
	Self-employed	34	34	31	31
	Unemployed	16	16	21	21
	Other	6	6	6	6
Monthly family income	≤Rs. 15,000	38	38	47	47
	Rs. 15,001-30,000	43	43	44	44
	≥ Rs. 30,001	19	19	9	9

**Table 2:** Effectiveness of garlic clove on blood pressure reduction (N=200)

**Parameter**	**Group**	**Pre-test Mean±SD**	**Post 7 days Mean±SD**	**Post 15 days Mean±SD**	**Mean Difference**	**Paired t-test**	**p-value**
Systolic BP (mmHg)	Interventional (n=100)	152.34±8.76	138.56±7.45	128.45±6.23	23.89	18.45	<0.001***
	Control (n=100)	151.89±8.92	150.23±8.67	149.78±8.54	2.11	1.34	NS
Diastolic BP (mmHg)	Interventional (n=100)	96.78±6.45	87.23±5.78	82.34±5.12	14.44	22.67	<0.001***
	Control (n=100)	95.67±6.89	94.89±6.76	94.45±6.58	1.22	0.98	NS

**Table 3:** Distribution of blood pressure categories in interventional group (n=100)

**BP Category**	**Pre-test n (%)**	**Post 7 days n (%)**	**Post 15 days n (%)**
Normal (<120/80)	0 (0.0)	0 (0.0)	0 (0.0)
Elevated (120-129/<80)	0 (0.0)	0 (0.0)	17 (17.0)
Stage 1 HTN (130-139/80-89)	0 (0.0)	56 (56.0)	83 (83.0)
Stage 2 HTN (≥140/≥90)	100 (100.0)	44 (44.0)	0 (0.0)
